# New and Confirmed Foci of Tick-Borne Encephalitis Virus (TBEV) in Northern Germany Determined by TBEV Detection in Ticks

**DOI:** 10.3390/pathogens11020126

**Published:** 2022-01-21

**Authors:** Anna-Katharina Topp, Andrea Springer, Gerhard Dobler, Malena Bestehorn-Willmann, Masyar Monazahian, Christina Strube

**Affiliations:** 1 Institute for Parasitology, Centre for Infection Medicine, University of Veterinary Medicine Hannover, 30559 Hannover, Germany; anna-katharina.topp@tiho-hannover.de (A.-K.T.); andrea.springer@tiho-hannover.de (A.S.); 2 National Reference Laboratory for TBEV, Bundeswehr Institute of Microbiology, 80937 Munich, Germany; gerharddobler@bundeswehr.org (G.D.); malena1bestehornwillmann@bundeswehr.org (M.B.-W.); 3 The Governmental Institute of Public Health of Lower Saxony (NLGA), 30449 Hannover, Germany; masyar.monazahian@nlga.niedersachsen.de

**Keywords:** tick-borne encephalitis, tick-borne diseases, vector-borne diseases, *Ixodes ricinus*, tick, microfocus, public health

## Abstract

Tick-borne encephalitis (TBE) is a tick-transmitted, virus-induced neurological disease with potentially fatal outcomes in humans and animals. Virus transmission takes places in so-called tick-borne encephalitis virus (TBEV) microfoci, which constitute small areas of sustained virus circulation. In southern Germany, TBEV has been endemic for decades; however, a northward expansion of risk areas, based on disease incidence in the human population, has been observed in recent years. The present study investigated TBEV occurrence in questing ticks at eight locations in the federal state of Lower Saxony, northwestern Germany, chosen due to reported associations with human TBE cases (N = 4) or previous virus detection (N = 4). A total of 20,056 ticks were collected in 2020 and 2021 and tested for TBEV RNA in pools of ten nymphs or five adults by quantitative reverse transcription-PCR (RT-qPCR). Positive results were confirmed by RT amplification of the viral E gene. In total, 18 pools from five different sampling locations were positive for TBEV RNA. One previously unknown transmission focus was detected, while ongoing virus circulation was confirmed at the four further locations. Phylogenetic analysis showed that two different virus strains with different origins circulate in the locations identified as natural foci.

## 1. Introduction

Tick-borne encephalitis virus (TBEV) is regarded as the most important viral tick-transmitted pathogen in Europe, and may cause severe neurological disease (tick-borne encephalitis, TBE) in humans as well as in a number of animals [1]. TBEV belongs to the mammalian tick-borne group within the genus *Flavivirus* of the family Flaviviridae. To date, at least five TBEV subtypes have been described; the European (TBEV-Eu), the Siberian (TBEV-Sib), the Far Eastern (TBEV-FE) and the recently detected Baikalian (TBEV-Bkl) as well as the Himalayan subtype (TBEV-Him) [2,3]. Apart from their geographical distribution, these five subtypes also differ in clinical presentation [4]. The clinical course in humans, but also in horses and dogs, can vary from flu-like to fatal neurological involvement. In a study with 635 human TBE patients, 47% showed meningitis, 42% meningoencephalitis and 11% meningoencephalomyelitis [5]. A vaccine is available, and it is generally accepted that vaccination is an inevitable measure to reduce the number of infections, especially in endemic risk-areas [6].

TBEV is mainly transmitted via the bite of an infected tick, while transmission by raw milk products of viraemic ruminants plays a minor epidemiological role [7]. In Europe, *Ixodes ricinus* acts as the main vector of TBEV-Eu, although the vector capacity of *Dermacentor reticulatus* has also been shown [8]. Ticks become infected when feeding on viraemic vertebrate hosts, predominantly small rodents and shrews, or non-viraemically via co-feeding of infected and non-infected ticks. The virus then persists through the tick’s lifecycle by transstadial and, though probably rarely, also transovarial transmission [9].

In contrast to many other tick-borne pathogens which are widespread in the tick population, TBEV circulates between small mammals and ticks in geographically restricted “microfoci”. These constitute small areas, sometimes only covering some 50 × 50 m, where continuous virus transmission takes place and which are located within a larger area of up to 1 km or more in diameter, where TBEV-positive ticks can be detected due to dispersal via rodents or other mammals [10]. The prevalence of TBEV in questing *I. ricinus* in these areas is rather low. As ticks are often pooled prior to TBEV testing, minimal infection rates (MIRs) are usually reported. In previous studies in Europe, MIRs in TBEV microfoci ranged from 0.45% in nymphs to 1.05% in adult ticks [11,12,13,14,15]. Nevertheless, there are also studies estimating TBEV prevalence within the transmission areas by testing ticks individually. In such a study from northeastern Germany, TBEV prevalence amounted to 2.4% [16], and to 6.4% in another study from Romania [17]. The low prevalence in the tick population also hampers investigation of the temporal stability of TBEV microfoci [13]. Nevertheless, the disappearance of microfoci has been described [18].

Despite these low MIR/prevalence values, the incidence of TBE is increasing in Europe and a geographical spread of the virus to previously non-endemic areas is evident [19,20,21]. In Germany, the National Institute of Public Health (Robert-Koch-Institut) classifies a district as an official risk area if the five-year clinical incidence in humans significantly exceeds 1/100,000 inhabitants. From 2001–2018, 89.0% of all human cases occurred in the German southernmost federal states of Baden-Wuerttemberg and Bavaria [6] and, consequently, most of the official 169 risk areas are located in these two federal states [22]. Nevertheless, TBEV is obviously spreading northwards, since more and more districts in other federal states have been declared risk areas since 2010 [23,24,25]. Furthermore, TBE cases also occur outside of official risk areas and virus detection in ticks and mammals indicates that virus circulation may be underestimated in non-risk areas [11,26]. Vaccination coverage in the German population is rather low, compared, e.g., to Austria where nearly 82% of inhabitants are vaccinated against TBEV [27]. A recent study estimated the vaccination coverage in Germany to range between a minimum of 10% in the northern Free and Hanseatic city of Hamburg and a maximum of 52% in the federal state Baden-Wuerttemberg. Due to the fact that approximately 50% of the population in endemic risk areas is unprotected, awareness of TBEV should be increased [28].

Although only one district (“Emsland”) in the northern German federal state of Lower Saxony is currently classified as an official risk-area, several human TBE cases have been reported in other districts of Lower Saxony in the recent past [24], leading to the assumption that there are as yet unidentified microfoci. The aim of the present study was to detect such unknown TBEV microfoci, based on their association with clinical cases, and to phylogenetically characterize the obtained virus isolates. Furthermore, the temporal stability of previously known microfoci in Lower Saxony [11,29] was investigated.

## 2. Results

### 2.1. TBEV Detection

**Figure 1:**
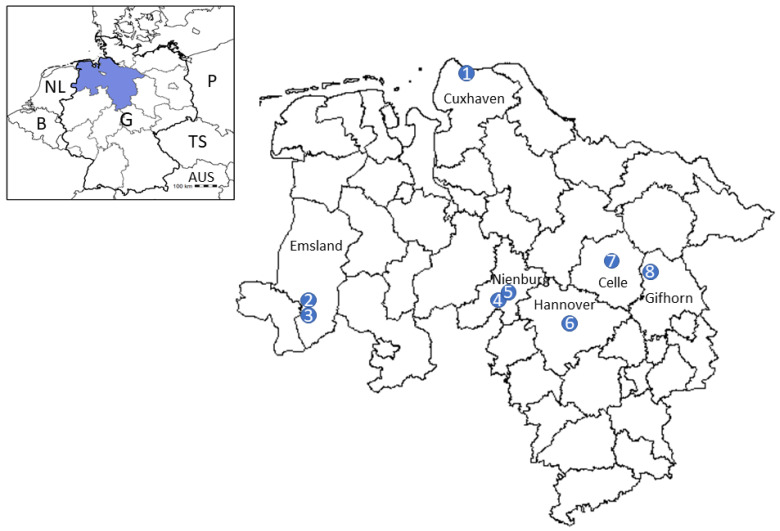
Geographical location of the eight sampling areas within the Northern German federal state Lower Saxony. (1) Wingst (district of Cuxhaven), (2) Lingen East (district of Emsland), (3) Lingen West (district of Emsland), (4) Lake “Die Rolle” (district of Nienburg), (5) Rauher Busch (district of Nienburg), (6) Barsinghausen/Mooshuette (district of Hannover), (7) Celle (district of Celle), (8) garden near Flettmar (district of Gifhorn). The small map shows the location of Lower Saxony within Germany. Abbreviations: G: Germany, NL: The Netherlands, B: Belgium, AUS: Austria, TS: Czech Republic, P: Poland.

In total, 20,056 questing ticks were collected at eight different locations in Lower Saxony (Figure 1) during April, May and September of 2020 as well as April and May 2021 (Table 1). Of these, 16,184 (80.7%) were nymphs and 3872 (19.3%) adult ticks. All collected ticks were morphologically identified as ticks of the *Ixodes ricinus*/*inopinatus* complex.

**Figure 2:**
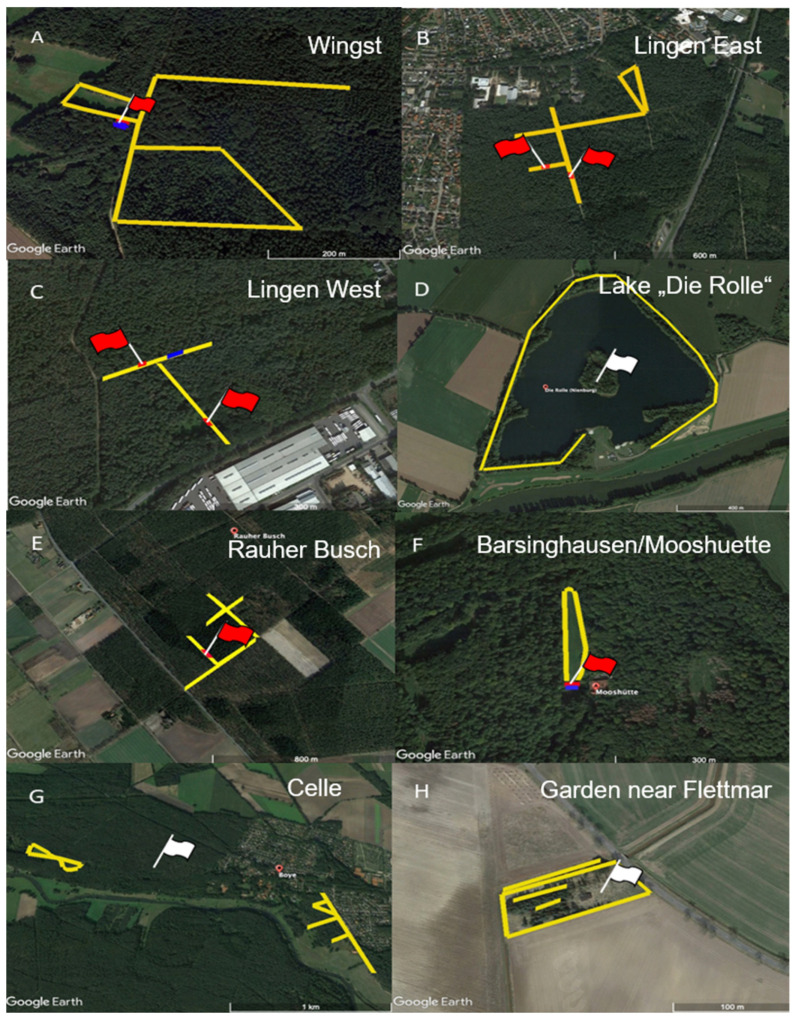
Tick sampling locations: (**A**) Wingst (district of Cuxhaven, 53°43′45.42″ N, 9°2′38.67″ E), (**B**) Lingen East (district of Emsland, 52°30′13.32″ N, 7°20′0.68″ E), (**C**) Lingen West (district of Emsland, 52°30′17.20″ N, 7°16′40.42″ E), (**D**) Lake “Die Rolle” (district of Nienburg, 52°38′15.69″ N, 9°10′25.99″ E), (**E**) Rauher Busch (district of Nienburg, 52°32′36.00″ N, 8°52′41.00″ E), (**F**) Barsinghausen/Mooshuette (district of Hannover, 52°19′10.31″ N, 9°23′58.74″ E), (**G**) Celle (district of Celle, 52°38′13.18″ N, 10°1′40.07″ E), (**H**) garden near Flettmar (district Gifhorn, 52°31′23.49″ N, 10°19′54.55″ E). Ticks were sampled along the tracks marked in yellow. Virus detection sites of the current study are shown in red; previous detections at locations C, E and H are marked in blue. Coordinates refer to the virus detection site (red flags) or the central point of the flagging area (white flags), respectively. Images created with Google Earth Pro version 7.3.4. 8248.

Of the 2416 examined pools containing up to ten nymphs or five adult ticks, respectively, eighteen (0.75%) pools from four locations were positive for TBEV-RNA by quantitative reverse transcription-PCR (RT-qPCR) (Table 2). A first-time virus detection was successful at the location “Lingen East” (district “Emsland”) with one positive pool in 2020 and seven positive pools in 2021, which were all collected from the same 200 m stretch of path (Figure 2B). The MIR in nymphs at this location amounted to 0.36% (7/1918) and the MIR ofadult ticks to 0.33% (1/304). Furthermore, virus detection was also successful at the second location in Lingen (“Lingen West”), with seven RT-qPCR-positive pools and MIRs of 0.13% (3/2345) in nymphs and 0.51% (4/791) in adult ticks. TBEV was previously detected at this location in 2019 (unpublished results), however, at a spot approximately 200 m away from the collection site of the positive ticks in 2020 (Figure 2C).

Additionally, continuous TBEV circulation was confirmed with one positive pool each at the locations “Barsinghausen/Mooshuette” (district Hannover), “Rauher Busch” (district Nienburg) and “Wingst” (district Cuxhaven), where the virus had already been detected in 2018 [11] and 2008/2009 [29]. The collection sites matched those of previous years at “Barsinghausen/Mooshuette” (Stefanie Becker, personal communication) and “Wingst” (unpublished data), whereas no information on the precise collection site at “Rauher Busch” in previous years was available. The respective MIRs are shown in Table 2.

### 2.2. Sequencing of the Viral E Gene and Phylogenetic Analysis

**Figure 3:**
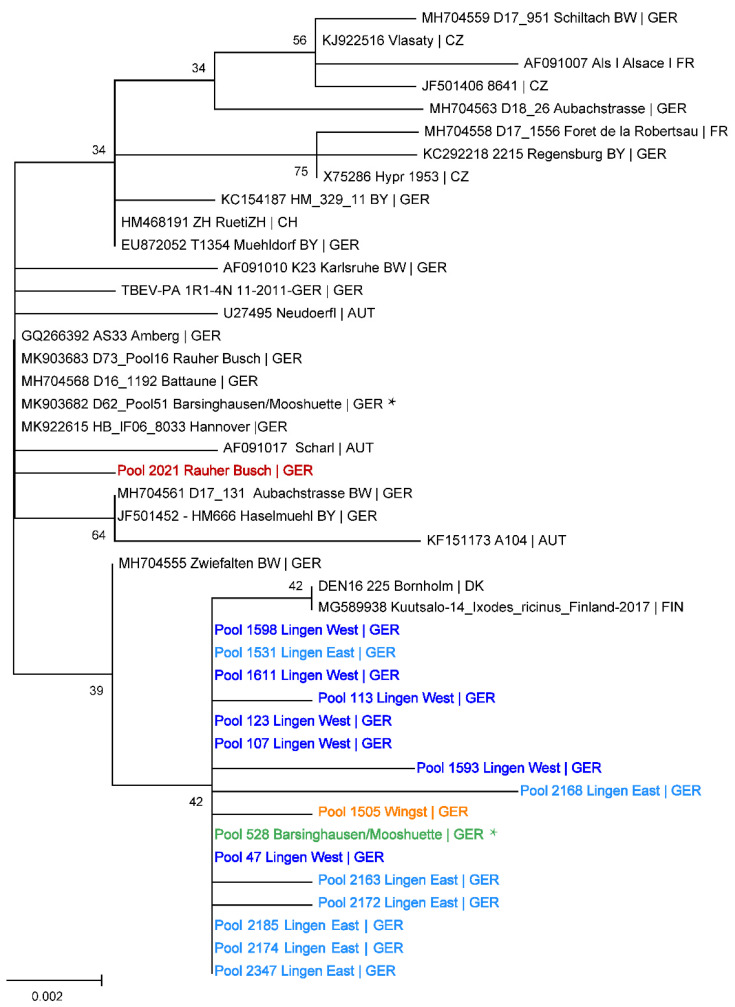
Maximum likelihood phylogenetic tree of the E gene sequences of TBEV virus strains generated in the present study (coloured according to sampling site: light blue, “Lingen East”; dark blue, “Lingen West”; green, “Barsinghausen/Mooshuette”; orange, “Wingst”) with other annotated TBEV sequences. Bootstrap support, i.e., the percentage of trees in which the associated taxa clustered together is shown next to the branches. Note the two different TBEV strains at the location Barsinghausen/Mooshuette (marked with asterisks).

Viral E gene sequences (1488 bp) of seventeen positive pools were generated (accession nos. OL743223-OL743239). In NCBI blast (www.blast.ncbi.nlm.nih.giv/Blast.cgi, accessed on 15 August 2021), the sequences isolated from Lingen East, Lingen West, Wingst and Barsinghausen/Mooshuette showed a high nucleotide identity (>99%) to each other and to the TBEV strain Kuutsalo-14_Ixodes_ricinus_Finland-2017 (98.99–99.70%) (accession no. MG589938; Figure 3). The strain from Rauher Busch was phylogenetically closely related to TBEV strains from Saxony, Germany (Battaune; accession no. MH704568), and to strains previously isolated from Rauher Busch (accession no. MK903683) as well as Barsinghausen/Mooshuette (accession no. MK903682).

### 2.3. Virus Cultivation

Virus cultivation was successful from six pools collected at the location “Lingen East” in 2021, whereas the virus could not be cultivated from the remaining 12 positive pools.

## 3. Discussion

The distribution of TBE in Germany is so far not well understood. While in large parts of southern Germany TBE is an endemic disease with high case numbers, in northern Germany only sporadic human cases are observed. In order to better understand this epidemiological pattern of TBEV in the northern federal state of Lower Saxony, the aim of this study was to describe previously unknown TBEV microfoci in areas where human TBE cases were reported, to analyse the phylogeny of the circulating TBE strains and to investigate whether virus circulation is still ongoing in previously detected microfoci or was only a temporary transmission. First-time virus detection in questing ticks was successful at one out of four investigated locations, namely, in “Lingen East” in the district of Emsland, the only official risk area in Lower Saxony. In addition, continuing virus circulation was confirmed at four further locations, one of them (“Lingen West”) located at a distance of only approximately 3.8 km from “Lingen East”, although separated by a federal highway as well as the river Ems and the Dortmund–Ems Canal. A direct spread of the virus from one microfocus to the other via tick-infested or viraemic wild mammals or birds seems probable [30]. Roe deer and wild boar, for example, can cover distances of up to 40 km in a short time [31].

The remaining areas, where continuous virus circulation was detected, are situated in districts not currently defined as risk areas. Nevertheless, autochthonous human TBE cases have been reported in all of these districts [23,24,25]. While initial TBEV detection at the locations “Lingen West” (unpublished data), “Barsinghausen/Mooshuette” and “Rauher Busch” occurred in 2018/2019 [11], TBEV was detected in 2008/2009 at the location “Wingst” [29], more than ten years prior to the current study. This is in line with the fact that the first human TBE case in that district was already reported in 2004 [23] and underlines the long-term stability of this microfocus [16].

Disappearance of microfoci was suspected in other studies, e.g., in North Zealand in Denmark, where TBEV was detected in ticks in 2009, 2010 and 2011, but not in 2016 [32]. However, microfoci may also persist in a state of endemic latency [33] with a low number of infected ticks [16]. In the present study, virus detection was only successful in one of the two study years at most locations. This may be related to the lower number of collected ticks in 2021. The low level of tick activity can be attributed to the very cold spring of 2021, characterized by temperatures close to 0 °C and even days with snow cover in April [34]. Although temperatures increased in May 2021, tick activity remained low, resulting in a low sample size of ticks.

Generally, prevalence of TBEV in tick populations is very low, with reported MIRs varying from 0.1% to 5.0% [35]. The results of the present study are in line with this range, varying from 0.00–0.32% in nymphs and 0.00–0.51% in adult ticks at those locations where TBEV was found. Here, the highest MIRs in nymphs and adults were detected in the town of Lingen, where most TBE cases in Lower Saxony have been reported. Nevertheless, the MIRs of the current study are in the lower range of previously reported values, although comparability between different studies can be limited, as the size of the sampling area and the number of ticks per pool may influence MIR values. In addition, year-to-year variations in prevalence exist. In a microfocus in southern Germany, which has been continuously monitored from 2009 until 2018, the nymphal MIR over the whole period amounted to 0.45%, with annual values ranging from 0.09% in 2009 to 1.36% in 2015 [13]. Boelke et. al. [11], who detected TBEV at the locations “Barsinghausen/Mooshuette” and “Rauher Busch” for the first time in 2018, described MIRs of 0.45% for nymphs and 1.05% for adult ticks [11]. In contrast, considerably higher MIRs have also been reported, e.g., on the Swedish island of Torö, where a well-known microfocus is located. There, the MIR for nymphs amounted to 0.51% and to 4.48% for adult ticks [14]. It has to be kept in mind that MIRs may underestimate the true prevalence in the tick population, as it is generally assumed that only one tick per positive pool is infected [36].

As mentioned above, TBEV microfoci may be as small as 50 × 50 m [10]. In the current study, TBEV-positive ticks were found in the same small area as in the previous studies [11,29] at the locations “Wingst” (unpublished data) and “Barsinghausen/Mooshuette” (Stefanie Becker, personal communication). Unfortunately, no information on the precise collection site of TBEV-positive ticks at “Rauher Busch” in previous years was available. Therefore, and because a rather large area was sampled in both studies, it is not possible to conclude whether the detection site of the positive tick pool in the present study corresponds to the previous detection site or represents a second microfocus. Furthermore, rodents and other small mammals may carry infected ticks out of the microfocus. As only one positive tick pool was found in the current study, the exact location of the microfocus at “Rauher Busch” remains to be confirmed. The TBEV-positive tick pools at “Lingen West” were collected at a distance of nearly 200 m from the previous detection site (unpublished data). One out of ten pools (1/98 nymphs) was found to be positive there during tick sampling activities in 2019. The detection of TBEV at the same place in two subsequent years confirms a stable natural focus.

Successful cultivation of the virus from “Lingen East” demonstrated the presence of infective virus particles in the ticks. Unfortunately, virus cultivation was not successful for all positive pools in the current study, which may have been due to a low virus load and to the sample transport between different laboratories.

The obtained TBEV sequences show that in the natural foci in Wingst/Cuxhaven as well as Lingen/Emsland a virus is circulating which is phylogenetically closely related to TBEV strains from the Danish island of Bornholm and the island of Kuutsalo, Finland. This distribution implies a spread by bird migration. This line, Kuutsalo, Finland–Bornholm–Cuxhaven–Lingen, represents one of the classic bird migration routes from northern Europe to southwestern Europe. Data show that birds may carry ticks when migrating from north to south and that these ticks might be infected with TBEV [37]. The isolate from Barsinghausen/Mooshuette found in the present study also clustered with these sequences, although a phylogenetically different isolate was detected at the same site in a previous study [11]. This implies that two different strains are circulating at this location, similar to findings from TBEV foci in southern Germany [31].

The isolate from Rauher Busch/Nienburg described here is genetically different from the Lingen strain cluster, but closely related to a previous isolate from this location [11] and to a TBEV strain found in northern Saxony (Battaune). Earlier data showed also a close phylogenetic relationship with Polish TBEV strains [38]. The fact that the TBEV strain in northern Saxony seems to be maintained in a natural transmission cycle in *Dermacentor reticulatus* is of special interest as this tick species is currently expanding its range in Germany [39], probably spreading from east to west, and has meanwhile reached Lower Saxony where these TBE strains were detected. Although *D. reticulatus* was not detected at the described TBEV foci, these observations might imply that a common factor is responsible for the spread of *D. reticulatus* and TBEV strains from this genetic clade. As *D. reticulatus* is not found on birds, this might be a first example of long distance spread of TBEV by terrestrial animals. However, more eco-epidemiological studies are necessary to reveal the mode of spread of this TBEV clade over long distances from Poland to Lower Saxony.

Unfortunately, detection of the presumptive TBEV transmission foci at the locations “Lake ‘Die Rolle’”, “garden near Flettmar” and “Celle” was not successful. Tick activity at the locations “Lake ‘Die Rolle’” and “garden near Flettmar” was low, resulting in a comparatively low number of collected ticks. These locations represented small, isolated patches of vegetation within an agricultural landscape, which may be one reason for the low tick abundance. Interpretation of these negative results, given the low sample size, is not possible. However, although the TBE patients reported that they presumably acquired the virus-transmitting ticks at these locations, it remains questionable whether this was really the case given the low tick abundance. A tick bite is often only discovered after a few days, and it may be difficult for the patient to narrow down the exact location [10]. In contrast, a large number of ticks was collected at the location “Celle”, but no TBEV was detected. The TBE patient in this case specified a rather large area (nearly 1.6 km^2^) in which the vector tick presumably could have been acquired. Consequently, tick sampling was spread over this large area. It remains possible that the microfocus was not within that collecting area, or that the number of ticks collected from the microfocus within the larger area was too low for successful virus detection, given the low TBEV prevalence within microfoci discussed above.

## 4. Materials and Methods

### 4.1. Tick Sampling

Questing ticks were sampled by the flagging method at eight locations in Lower Saxony, Germany (Figure 1). Four sampling locations (“Lingen East” (district Emsland), Lake “Die Rolle” (district Nienburg), “Celle” (district Celle) and “garden near Flettmar” (district Gifhorn)) were chosen based on movement patterns of human TBE patients prior to disease onset, reported to the Governmental Institute of Public Health of Lower Saxony. In the case of “Lingen East” and “Celle”, more than one clinical TBE case was presumably associated with the chosen sampling area. The locations “Lingen West” (district Emsland, unpublished data), “Rauher Busch” (district Nienburg [11]), “Barsinghausen/Mooshuette” (district Hannover [11]) and “Wingst” (district Cuxhaven [29]) were chosen based on virus detection in questing ticks in previous studies.

Tick collection took place in April, May of 2020 and 2021 and September 2020 by dragging a 1 m^2^ white cotton cloth over the low vegetation and checking for ticks every five meters. Figure 2 illustrates the sampling strategy at each location. To increase the chance of sampling infected ticks, flagging was conducted along paths presumably used by the TBE patients, rather than randomly in the forested area. In the case of “garden near Flettmar” (district of Gifhorn), the location was a private garden and flagging was conducted in the entire suitable tick habitat within this garden (Figure 2H).

Collected *Ixodes* ticks were identified based on morphological keys by Estrada-Peña et al. [40] and pooled according to stage and gender, so that each pool contained a maximum of five adults or ten nymphs. Until further examination, the pools were stored at −80 °C.

### 4.2. RNA-Isolation and RT-qPCR

To each tick pool, 500 µL Minimal Eagle’s Medium (MEM) and three steel beads were added prior to homogenization in a Precellys^®^ 24 instrument (3 × 6000 rpm for 30 sec.; PEQLAB Biotechnologie GmbH, Erlangen, Germany). Total RNA was extracted from 100 µL of the homogenate using the NucleoSpin Virus Kit (Macherey-Nagel, Dueren, Germany), following the manufacturer’s instructions. Elution of viral RNA was conducted with 130 µL RNase-free water in two steps (70 µL and 60 µL).

Extracted RNA was screened for TBEV-RNA by RT-qPCR, following the protocol developed by Schwaiger and Cassinotti [41]. As positive control, 10 µL TBEV-RNA of the Austrian Neudoerfl strain (U27495.1) was used, and negative controls included 10 µL RNase-free water as template. Each of the 2416 pools was tested in duplicate, and positive pools were tested for a second time to exclude false positive results. Minimum infection rates were calculated under the assumption of only one positive tick per pool, i.e., by dividing the number of positive pools by the number of total ticks.

### 4.3. Amplification and Sequencing of the Viral E Gene

RT-qPCR positive samples were subjected to RT amplification of the viral E gene at the Institute for Parasitology, University of Veterinary Medicine Hannover, or at the Bundeswehr Institute of Microbiology, Munich. To this end, the primer pairs TBE-885/TBE-c2751a/b [42] were used in a conventional RT-PCR. Amplicons generated in Munich were purified using a QIA quick PCR Purification Kit (Qiagen, Hilden, Germany) and sequenced with the outer primers and an additional internal sequencing primer (TBE-c1648) as described in [42] (GATC, Eurofins Genomics, Ebersberg, Germany). The amplicons generated in Hanover were purified with the GeneJet Purification Kit (Thermo Fisher Scientific, Waltham, MA, USA), following the manufacturer’s instructions. Products were ligated into a TOPO-TA™ vector and inserted into chemically competent *E. coli* cells (Invitrogen™ OneShot™ TOP10) using the TOPO™ TA Cloning™ Kit (all Thermo Fisher Scientific, Waltham, MA, USA). Purified plasmids (NuceloSpin™ Plasmid DNA Purification Kit, Macherey-Nagel, Germany) were sent for custom Sanger-sequencing (Microsynth Seqlab, Göttingen, Germany) with primers TBE-c1648 [42] as well as primers M13 and M13r. The obtained sequences were assembled using Clone Manager software (v. 9.3, Sci Ed Software LLC, Westminster, CO, USA) and compared to publicly available TBEV sequences using NCBI blast (www.blast.ncbi.nlm.nih.gov/Blast.cgi, accessed 15 August 2021).

### 4.4. Phylogenetic Analysis

Sequence data were processed using the program Geneious Prime (Biomatters, Ltd., Auckland, New Zealand) or Clone Manager (v. 9.3, Sci Ed Software LLC, Westminster, CO, USA). Consensus sequences were derived by performing a de novo assembly using the three chromatograms of each positive sample. Nucleotides with an estimated error higher than 1% were trimmed. Subsequently, the sequences were cut to 1488 bp, the exact sequence of the envelope gene. A ClustalW alignment with additional E genes from selected isolates or from the NCBI data base was performed and a phylogenetic tree was generated using the maximum likelihood method (1000 bootstrap replicates) and a discrete Gamma distribution with evolutionarily invariable sites (G+I) in Mega v. X [43].

### 4.5. Virus Cultivation

Homogenized tick suspensions were diluted 1:5 and 1:25 in MEM (Invitrogen, Karlsruhe, Germany), containing 3% fetal calf serum (FCS, Invitrogen, Karlsruhe, Germany) and antibiotic–antimycotic solution (ABAM, Invitrogen, Karlsruhe, Germany) at 10-fold concentration as recommended. Then, 0.5 mL of the diluted tick suspensions were inoculated on A549 cells (German Collection of Microorganisms and Cell Cultures, DSMZ, Braunschweig, Germany) in T25 cell culture flasks (Thermo Scientific, Dreieich, Germany). After inoculation for 60 min at 37 °C, the inoculate was removed and the cells were washed three times with MEM + 10-fold ABAM. Finally, 5 mL of MEM + 10-fold ABAM + 3% FCS were added, and cells were incubated for 5 days at 37 °C. After 5 days, 140 µL of the supernatant were removed and tested for TBEV RNA by RT-qPCR, as described earlier [42]. In positive ticks, the original tick suspension was used for sequencing the TBEV E gene directly from the tick as described above.

## 5. Conclusions

The detection of a previously unknown TBEV transmission focus and confirmation of ongoing virus circulation in several areas in the northern German federal state of Lower Saxony underlines the TBE risk outside of the German regions classified as official risk areas. Therefore, raising public awareness with regard to vaccination and increased surveillance efforts are required. For example, screening of sentinel animals for TBEV antibodies may allow detection of further areas of virus circulation. Continuous monitoring of TBE transmission foci allows more knowledge to be gained regarding their temporal development.

## Figures and Tables

**Table 1:** Number of collected ticks per sampling location in 2020 and 2021.

Location	District	2020			2021			Total
		Total	Nymphs	Adult Ticks	Total	Nymphs	Adult Ticks	
Wingst ^1^	Cuxhaven	2902	2441	461	691	555	136	3593
Lingen East ^2^	Emsland	1700	1486	214	522	432	90	2222
Lingen West ^1^	Emsland	2652	1880	772	484	465	19	3136
Lake “Die Rolle” ^2^	Nienburg	862	645	217	145	90	55	1007
Rauher Busch ^1^	Nienburg	3124	2856	268	880	718	162	4004
Barsinghausen/Mooshuette ^1^	Hannover	1856	1446	410	319	209	110	2175
Celle ^2^	Celle	3160	2405	701	636	140	496	3742
Garden near Flettmar ^2^	Gifhorn	163	55	108	14	9	5	177

^1^ Previous virus detection. ^2^ Newly selected locations due to association with clinical TBE case(s).

**Table 2:** Minimal infection rates (MIR) ^1^ for each sampling location.

Location	District	No. of Collected Ticks	No. of Pools	Positive Pools (Nymphs/Adults)	Positive Pools in 2020 (Nymphs/Adults)	Positive Pools in 2021 (Nymphs/Adults)	Total MIR Nymphs (%)	Total MIR Adults (%)
Wingst ^2^	Cuxhaven	3593	401	1 (1/0)	1 (1/0)	0 (0/0)	0.03	0.00
Lingen East ^3^	Emsland	2222	249	8 (7/1)	1 (0/1)	7 (7/0)	0.36	0.33
Lingen West ^2^	Emsland	3136	367	7 (3/4)	7 (3/4)	0 (0/0)	0.13	0.51
Lake “Die Rolle” ^3^	Nienburg	1007	98	0 (0/0)	0 (0/0)	0 (0/0)	0.00	0.00
Rauher Busch ^2^	Nienburg	4004	446	1 (1/0)	0 (0/0)	1 (1/0)	0.03	0.00
Barsinghausen/Mooshuette ^2^	Hannover	1856	283	1 (0/1)	1 (0/1)	0 (0/0)	0.00	0.19
Celle ^3^	Celle	3742	654	0 (0/0)	0 (0/0)	0 (0/0)	0.00	0.00
Garden near Flettmar ^3^	Gifhorn	177	30	0 (0/0)	0 (0/0)	0 (0/0)	0.00	0.00

^1^ Minimal infection rates were calculated under the assumption of only one positive tick per pool, i.e., by dividing the number of positive pools by the number of total ticks. ^2^ Previous virus detection. ^3^ Newly selected locations due to association with clinical TBE case(s).

## Data Availability

Data supporting reported results is contained within the article.
